# Draft Genome Sequence of *Desulfosporosinus* sp. Strain Sb-LF, Isolated from an Acidic Peatland in Germany

**DOI:** 10.1128/MRA.00428-19

**Published:** 2019-07-18

**Authors:** Bela Hausmann, Petra Pjevac, Martin Huemer, Craig W. Herbold, Michael Pester, Alexander Loy

**Affiliations:** a Centre for Microbiology and Environmental Systems Science, Department of Microbiology and Ecosystem Science, Division of Microbial Ecology, University of Vienna, Vienna, Austria; b Joint Microbiome Facility of the Medical University of Vienna, University of Vienna, Vienna, Austria; c Department of Laboratory Medicine, Medical University of Vienna, Vienna, Austria; d Department of Microorganisms, Leibniz Institute DSMZ—German Collection of Microorganisms and Cell Cultures, Braunschweig, Germany; e Institute of Microbiology, Technical University of Braunschweig, Braunschweig, Germany; f Austrian Polar Research Institute, Vienna, Austria; Indiana University, Bloomington

## Abstract

*Desulfosporosinus* sp. strain Sb-LF was isolated from an acidic peatland in Bavaria, Germany. Here, we report the draft genome sequence of the sulfate-reducing and lactate-utilizing strain Sb-LF.

## ANNOUNCEMENT

Dissimilatory sulfate reduction is an important anaerobic carbon mineralization process in peatlands and mitigates the production of the greenhouse gas methane (1). Members of the genus *Desulfosporosinus* are present in wetlands worldwide but typically at very low relative abundances (2). Despite its low abundance and near-zero growth, “*Candidatus* Desulfosporosinus infrequens” SbF1 was a major driver of sulfate reduction in experimental microcosms established with acidic soil from the Schlöppnerbrunnen II fen (Bavaria, Germany) (2–4). Here, we report the draft genome sequence of *Desulfosporosinus* sp. strain Sb-LF, which was isolated from the same peatland.

Strain Sb-LF was enriched and isolated as described previously under sulfate-reducing conditions, with l-lactate as the carbon source (5, 6). Briefly, 10 ml of culture was grown to stationary phase on freshwater minimal medium (6) amended with sulfate (5 mM) and l-lactate (10 mM) and harvested by centrifugation. Genomic DNA was isolated using the DNeasy PowerSoil kit (Qiagen), and sequencing libraries were prepared using the Nextera XT kit (Illumina) and sequenced on the Illumina HiSeq 2000 platform, yielding 55 million 120-bp paired-end reads. The reads were inspected with FastQC (http://www.bioinformatics.babraham.ac.uk/projects/fastqc/) and quality trimmed at a Phred quality score of 10 using the BBDuk function of BBMap v34.94 (https://sourceforge.net/projects/bbmap/) and retaining 99.89% of the reads. Quality-trimmed reads were assembled using SPAdes v3.6.2 (7) and, subsequently, iteratively (*n* = 5) reassembled with SPAdes v3.11.1 using contigs of >1 kb from the previous assembly as “trusted contigs” for input and iterating kmers from 11 to 121 in steps of 10. In total, 75.08% of the quality-trimmed reads used for assembly could be unambiguously mapped back to the draft genome. The draft genome sequence consists of 30 scaffolds with a total size of 4,272,165 bp, a G+C content of 42.5%, and an *N*_50_ value of 337,508 bp. Based on CheckM (8), the completeness of the draft genome is 99.9%, with three duplicated single-copy marker genes.

Taxonomic placement of strain Sb-LF into the genus *Desulfosporosinus* was verified using a concatenated alignment of 22 unique marker genes using the GTDB toolkit (9) and IQ-TREE (10). The most similar genome was that of *Desulfosporosinus* sp. OL (GenBank assembly accession number GCA_001936615.1) with an average nucleotide identity (ANI) of 81% (alignment fraction, 0.44) (11), well below the intraspecies threshold of 96.5% (12). Average amino acid identities (AAI) were 74% to 82% to *Desulfosporosinus* species (compared to all genomes listed at NCBI, November 2018) and 78% to “*Candidatus* Desulfosporosinus infrequens” SbF1 (13), which indicates that strain Sb-LF represents a novel *Desulfosporosinus* species.

The genome was annotated using Rapid Annotations using Subsystems Technology (RAST) (14) and the NCBI Prokaryotic Genome Annotation Pipeline. The genome carries 3,924 coding sequences (CDSs), 13 rRNAs, 92 tRNAs, and 7 noncoding RNAs (ncRNAs). Genes for the canonical pathway for dissimilatory sulfate reduction with adenylyl-sulfate reductase (*aprBA*) and dissimilatory sulfite reductase (*dsrAB*) as key enzymes are present. A trimeric sulfite reductase (*asrABC*), as encoded in other members of the genus *Desulfosporosinus* (4), was not detected. The genome contains multiple identified copies of lactate dehydrogenase genes for putative l-lactate degradation.

### Data availability.

This whole-genome shotgun project has been deposited at DDBJ/ENA/GenBank under the BioProject number PRJNA529085 and accession number SPQR00000000. The version described in this paper is version SPQR01000000.
